# Study of Flow Stress Models and Ductile Fracture Criteria for CHN327 Nickel-Based Superalloy

**DOI:** 10.3390/ma16062232

**Published:** 2023-03-10

**Authors:** Yufeng Xia, Wenbin Yang, Yingyan Yu, Haihao Teng, Qian Cheng

**Affiliations:** 1 School of Materials Science and Engineering, Chongqing University, Chongqing 400044, China; 2 Chongqing Key Laboratory of Advanced Mold Intelligent Manufacturing, School of Materials Science and Engineering, Chongqing University, Chongqing 400044, China

**Keywords:** CHN327 alloy, flow stress, constitutive model, ductile fracture criteria

## Abstract

The plastic deformation behavior of a CHN327 nickel-based superalloy under temperatures ranging from 600 °C to 700 °C and strain rates ranging from 0.001 to 0.1 s^−1^ was investigated using uniaxial high-temperature tensile tests. The stress–strain curves obtained by the tests showed that the maximum stress decreased as the temperature increased, while it increased as the strain rate increased. Based on the extensive data obtained in the experiment, three constitutive models (Hollomon, Swift, and the modified Voce equation) were employed to predict the constitutive relation. It was found that the modified Voce equation had the highest correlation coefficient and the best prediction accuracy. Thereafter, in order to predict the fracture of CHN327 during high-temperature tensile deformation, five ductile fracture criteria (Freudenthal, C&L, Brozzo, Ayada, and the R&T model), and the modified Voce equation obtained were incorporated into the finite element software (DEFORM). According to the results, except for the C&L and Brozzo models, all of the other ductile fracture criteria (DFCs) were suitable for predicting the damage distribution of the CHN327 alloy in tensile tests. For all of the DFCs considered, the R&T model provided the most accurate predictions, whose mean error was only 8.9%, far less than the values that other models predicted.

## 1. Introduction

Today, shortening the manufacturing cycle of the forging die, reducing the manufacturing cost, and extending the service cycle effectively are the research focuses of forging die development [1]. Traditional forging dies and casting forging dies are generally made of homogeneous materials, but once some areas fail, they cannot continue to be used, resulting in high costs, a long production cycle, and low material utilization [2]. To improve the situation, the surfacing method is being used to gradually repair the mold [3]. Sun [4] found that the service life of a forging die forging connecting rod after surface repair can reach 60% of the service life of the new die, while the repair cost is less than 10% of the cost of the new die, and the production cycle is also shortened by more than 60%. Liu et al. [5] used plasma spray welding technology to deposit Fe-Cr-C-Ni powder on the base metal steel and observed its wear resistance. S Lu et al. [6] used cast steel as the basal body of the forging die and strengthened the cavity through bimetal-gradient-layer surfacing, which effectively reduced the manufacturing cost of the forging die and increased the service life of the die greatly. However, when the large forging die manufactured by bimetal-gradient-layer surfacing technology was applied to the 800 MN hydraulic press (the largest one around the world) for the deformation of hard-to-deform materials, the high temperature and heavy load resulted in problems such as severe deformation and wear of the die surface layer, extremely short life time, high cost, etc., and it could only improve the situation to some extent [7].

Under these circumstances, our experimental team proposed an improved method based on a “fist-like” structure of forging dies based on bimetal-gradient-layer surfacing, aiming to solve the problems mentioned above. Through surfacing a relatively soft material (the “skin”) on the available forging die (the “bone”) made by bimetal-gradient-layer surfacing [8], the “fist-like” die is obtained. The “bone” can support the whole structure under a heavy load, and at the same time, the “skin” can bear the high temperature, which usually reaches 650 °C during hot forging. Considering that the service environment for forging dies is under high temperature and pressure, the selection of welding materials is quite important. The surfacing materials commonly used at home and abroad can be divided into three categories according to the composition: nickel-based alloys, cobalt-based alloys, and iron-based alloys. At present, iron-based alloy welding materials have been widely used because of their cheap price and excellent performance. Despite the fact that nickel-based and cobalt-based alloy welding materials are expensive, they have good high-temperature performance and are increasingly used in mold repair [9].

To ensure the long service life of the “fist-like” forging die, a nickel-based superalloy welding material, CHN327 alloy, was selected in this study as the “skin” layer. CHN327 alloy is a Ni70Drl15 heat-resistant alloy electrode with a low hydrogen coating that has excellent high-temperature stability, good wear resistance, and corrosion resistance [10]. Research investigations have been carried out on its microstructure and mechanical properties, but less attention has been paid to modeling the flow behavior and fracture failure [11]. Furthermore, understanding the failure mechanisms and modes of the “skin” layer material under high temperatures and heavy loads is also a key question that needed to be solved. To predict the whole deformation process more accurately, it is necessary to develop a damage failure model by conducting simulations and experiments, and quantifying the peak stress development and fracture behavior of the CHN327 alloy.

Nowadays, finite element analysis has become an important method for establishing damage failure models. Li et al. [12] provide insight into the root cause of the difference in theoretical DFC prediction through quantitative and qualitative assessment of the applicability and reliability of uncoupled and coupled criteria. Zhan et al. [13] embedded the C&L and Lemaitre criteria into the finite element model to predict the fracture of aluminum alloy in the spinning process and compared the results with the experimental results to verify the applicability of the above DFCs. Lou et al. [14] applied a physical mechanism-motivated macroscopic ductile fracture criteria into a numerical simulation, which accurately predicted the onset of ductile fracture for DP980 steel sheets in various loading conditions from shear to plane strain tension. For the “fist-like” forging die with a large cast steel substrate, its comprehensive performance is determined by the performance of the cast steel substrate and bimetal surfacing layer. In the process of use, the cast steel substrate does not contact the forging directly, and the working conditions are good. The bimetallic surfacing layer, especially the surface layer, bears high thermal stress and thermal cycling, which is the main source of failure [15]. However, there is little research on the damage failure mechanisms and the simulation of the surface layer of the forging die made by bimetal gradient surfacing [8]. Therefore, the research in this study has special significance in terms of scientific research and significant application prospects, which is very innovative.

In this study, by deducing and analyzing the extensive data obtained in tensile tests, the mathematical model of the relationship between the flow stress and deformation conditions of the CHN327 alloy was established. Meanwhile, combined with finite element analysis, the damage parameters based on the DFCs were obtained. Then, through a simulation and experiments, the DFCs that were suitable for high-temperature deformation of CHN327 alloy were established. The applicability of these criteria was evaluated in terms of the fracture strain in different samples, and the accuracy of the selected damage models was verified by comparing them with the results of tensile experiments.

## 2. Experimental Procedures

### 2.1. Materials

In this study, a nickel-based superalloy, the CHN327 alloy, was selected as the welding material to fabricate the “skin” strengthening layer of the “fist-like” hot forging die fabricated by bimetal-gradient-layer surfacing. The chemical composition of this alloy is listed in Table 1. The elements C, Cr, and Mo are the main solution-strengthening elements to improve the hardness, strength, and abrasive resistance of the alloy [16]. Furthermore, due to the high content of chromium, a high hardness chromium oxide film is formed on the surface of dies [17]. Compared with ferrous welding materials, CHN327 alloy welding materials have superior high-temperature properties as well as a moderate creep strength above 650 °C, and their strength and hardness will not decrease significantly at about 650 °C [7]. A piece of casting steel was selected as the matrix material. Ferrous welding electrodes RMD142 and RMD248 produced by Chongqing Jiepin Science & Technology Co., Ltd. (Chongqing, China) were used to fabricate the transition layers, and the chemical compositions of RMD142 and RMD248 are listed in Table 2 and Table 3.

### 2.2. Bimetal-Gradient-Layer Surfacing

**Figure 1:**
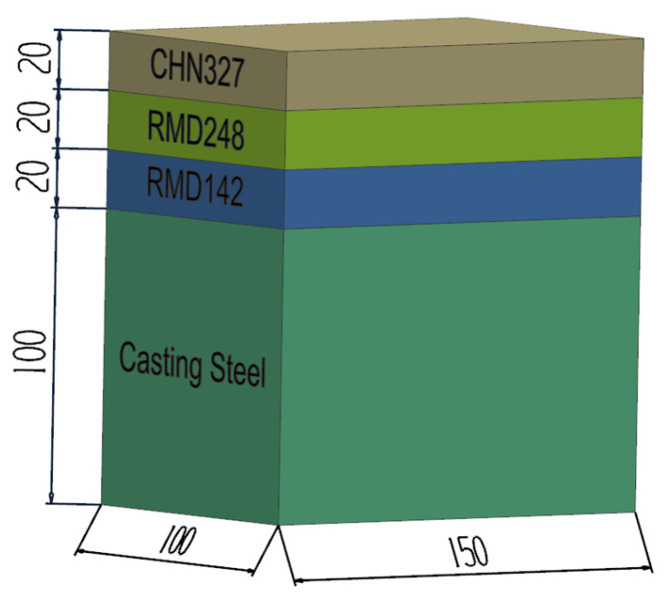
Schematic diagram and dimensions of sample (unit: mm).

The casting steel was heated to 450 °C by a resistance heading furnace and held isothermally for 2 h. Then, all the electrodes were heated to 250 °C and held isothermally for 1 h. Bimetal-gradient-layer surfacing was conducted by a ZX5-250 type DC welder with a welding speed of 3 mm/s, a welding current of 130 A, and a welding voltage of 26 V. CO_2_ and Ar gases with volume fractions of 20 and 80%, respectively, were employed for the protective atmosphere. The thickness of the welding pass was about 5 mm, and the weld interpass temperature was more than 300 °C. The heat treatment was tempering it at 550 °C for 1 h and cooling it in a furnace. The sample with the structure shown in Figure 1 was fabricated, which was the simple model used in the basic research of this study.

### 2.3. Tensile Test

**Figure 2:**
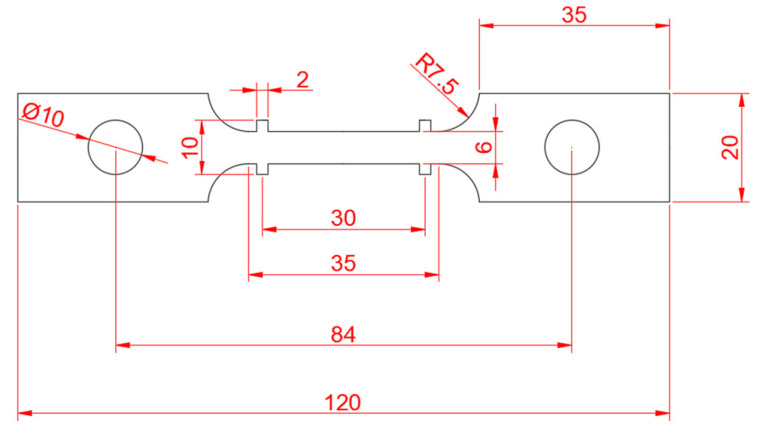
The sample size for the tensile (unit: mm).

**Figure 3:**
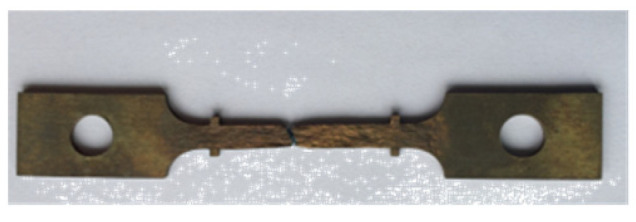
Samples after tension tests.

The fracture strain at different temperatures and strain rates was obtained by uniaxial isothermal tensile tests, which were conducted by using a WDW-100 universal testing machine equipped with a stove for heating. The geometry of the sheet samples is presented in Figure 2. They were designed according to the high-temperature tensile test method for metallic materials in GB/T 4338-1995 (the thickness was 2 mm). Each numbered experiment was conducted twice to ensure the accuracy and reliability of the experimental data. The tensile test was controlled by displacement, and the stretching process continued until the sample was broken. The sudden drop in the load magnitude during loading was identified as the moment of fracture. The strain at fracture was determined by measuring the elongation of the gauge. Figure 3 shows the morphology of the specimen after fracture. The fracture strains at different temperatures and strain rates are shown in Table 4 [18]. With the increase in temperature and the decrease in strain rate, the peak stress of the CHN327 alloy decreased, while the fracture strain showed no obvious change in the 600~700 °C temperature range and the strain rate from 0.001 to 0.1 s^−1^.

## 3. Establishment of Constitutive Model of Flow Behavior

### 3.1. Characteristics of True Stress—Strain Curves

**Figure 4:**
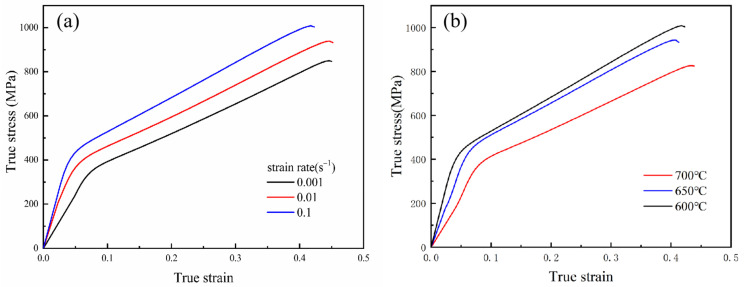
True stress—strain curves of CHN327 alloy at (**a**) different strain rates at 600 °C and (**b**) different temperatures at 0.1 s^−1^.

The true stress—strain data of the CHN327 alloy under different deformation conditions were obtained by tensile tests, as shown in Figure 4. The flow stress increased with decreasing temperature and increasing strain rate, the reason for which being that the effect of strain hardening is more obvious at a lower temperature and a higher strain rate [19]. Theoretically, at low temperatures, the internal energy, or activation energy, is lower and the atomic vibration is less, thus hindering the dislocation movement [20]. At high strain rates, dislocations accumulate rapidly, and the rheological softening time is limited, leading to an increase in the deformation resistance [21]. Additionally, the stress–strain curves under different deformation conditions have similar characteristics and can be divided into two stages. In the first stage, the rapid growth of dislocation density leads to work hardening, and the stress increases to a critical value approximately linearly with the increase in strain. In the second stage, the stress becomes less sensitive to strain, increasing slowly until the ultimate tensile strength is reached.

### 3.2. Classical Plastic Constitutive Equations

The flow stress at high temperatures is affected by the strain rate and temperature. Generally, the Arrhenius constitutive model considering strain compensation is employed to fit the stress–strain curve. However, the fitting accuracy is not ideal under moderate temperature conditions. According to the research results in [22], the upper limit range of the stress–strain curve can be described by a hardening model, such as the Holloman, Swift, and Voce equations.

One of the classical plastic constitutive equations characterizing the stress–strain curve of metal alloys is the Hollomon power law [23]:

(1) $$\sigma =K_{H}\varepsilon_{P}^{n_{H}}$$

where $K_{H}$ is the strength coefficient and $n_{H}$ is the strain hardening exponent.

Since the Hollomon equation has few parameters and is too simple, sometimes it cannot describe the hardening behavior of some materials well. Swift [24] proposed a pre-strain equation to describe the stress–strain characteristics of some materials more scientifically.

(2) $$\sigma =K_{S}{\left(\varepsilon_{P}+\varepsilon_{0}\right)}^{n_{s}}$$

where $n_{s}$ is the strain hardening exponent, $K_{S}$ is the strength coefficient, and $\varepsilon_{0}$ accounts for a possible pre-strain.

While both the Hollomon and Swift equations follow the power law, Voce [25] proposed an exponential function, which is obviously different from the power function, and it tends to be saturated under large strains. On this basis, Guo et al. [26] proposed a novel stress–strain function which is expressed as:

(3) $$\sigma =\sigma_{0}\left(1-e^{-b\varepsilon }\right)-k\varepsilon$$

Here, *σ* is the flow stress, *Ɛ* is the strain, and $\sigma_{0}$, b, and *k* are the material constants. In this equation, the flow curve is considered as a transient form of flow stress from a certain initial value to the saturation value corresponding to some equilibrium structures under a given strain rate and temperature [27].

Tensile stress–strain curves of the CHN327 alloy were analyzed by applying the commercial software OriginPro 2021. Table 5 gives all of the constitutive parameters and the predictive accuracy by Hollomon, Swift, and the modified Voce equations with full range fittings.

According to the research results in [26], as for the modified Voce equation, the material coefficients *b* and *k* are affected by the temperature and strain rate, and their formulas can be constructed by means of multiple linear functions. They are expressed as:

(4) $$b=b_{0}+b_{1}T+b_{2}\ln\dot{\varepsilon }+b_{3}T\ln\dot{\varepsilon }$$

(5) $$k=k_{0}+k_{1}T+k_{2}\ln\dot{\varepsilon }+k_{3}T\ln\dot{\varepsilon }$$

Here *b_i,_ k_i_, i* = 0, 1, 2, 3, are material constants.

Therefore, the effects of temperature *T* and strain rate on the material constants *b* and *k* were analyzed by applying the commercial software Originpro 2021. The fitting results showed that *b*_0_ = 488.06, *b*_1_ = −0.47, *b*_2_ = 68.33, *b*_3_ = −0.07, *k*_0_ = −6157.95, *k*_1_ = 5.05, *k*_2_ = −744.32, and *k*_3_ = 0.769.

### 3.3. Validation and Discussion

In order to evaluate and assess the performance of the three equations, the correlation coefficient (*R*) and absolute value of the average relative error (*AARE*) were quantified to evaluate the correlation between the fitted data and the experimental data. The closer the value *R* was to the value 1, the stronger the correlation was between the predicted data and the experimental data. *AARE* considers the error between each predicted value and the measured value, and is considered an unbiased quantity [28]. The statistical parameters *R* and *AARE* are introduced as [29]:

(6) $$R=\frac{\sum_{i=1}^{N}\left(E_{i}-\bar{E}\right)\left(P_{i}-\bar{P}\right)}{\sqrt{\sum_{i=1}^{N}{\left(E_{i}-\bar{E}\right)}^{2}\sum_{i=1}^{N}{\left(P_{i}-\bar{P}\right)}^{2}}}$$

(7) $$AARE\left(\%\right)=\frac{1}{N}\overset{N}{\underset{i=1}{\sum }}\left|\frac{P_{i}-E_{i}}{E_{i}}\right|\times 100\%$$

where $E_{i}$ and $P_{i}$ denote the experimental and predicted stresses, $\bar{E}$ and $\bar{P}\;$ describe the mean value of $E_{i}$ and $P_{i}$, respectively, and *N* presents the total number of observations.

**Figure 5:**
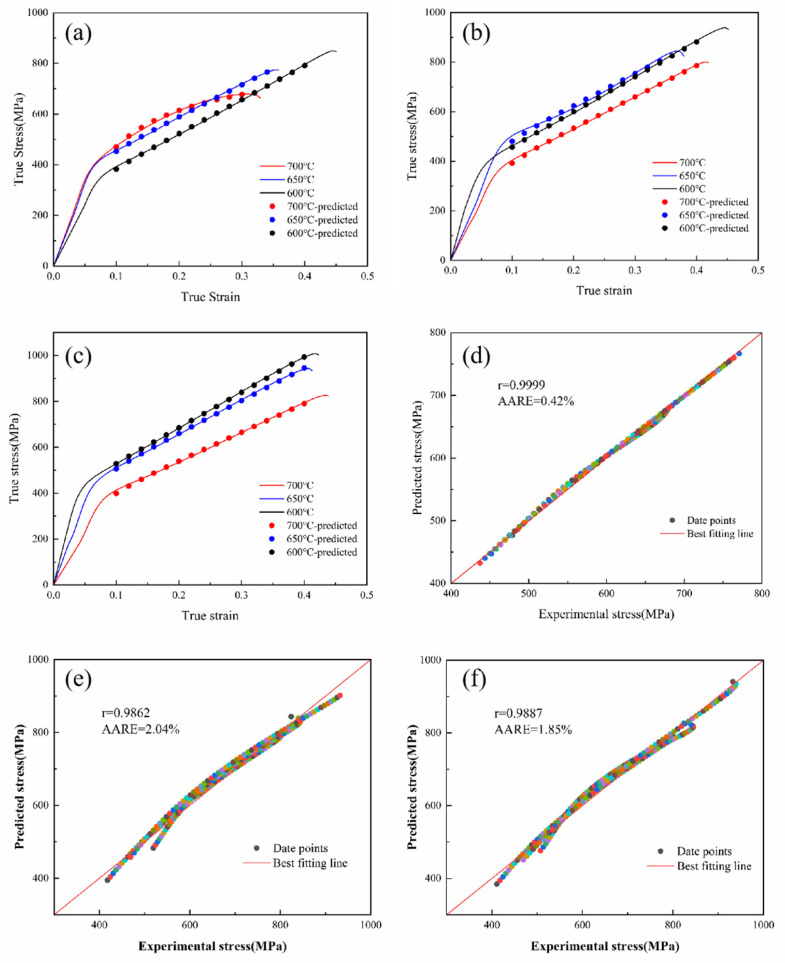
Comparison between predicted and experimental flow stress of CHN327 alloy at different temperatures and strain rates by the modified Voce equation: (**a**) 0.001 s^−1^; (**b**) 0.01 s^−1^; (**c**) 0.1 s^−1^; correlation between experimental and fitted flow stress values: (**d**) the modified Voce; (**e**) Hollomon; (**f**) Swift.

Figure 5 shows the correlation and *AARE* between the experimental and the predicted stresses for the CHN327 alloy obtained using the modified Voce, Hollomon, and Swift equations. Based on the comparison of the *R* and *AARE*, the order of priority was as follows: *R*_modified Voce_ > *R*_Swift_ > *R*_Hollomon_; *AARE*_modified Voce_ > *AARE*_Swift_ > *AARE*_Hollomon_. Among Hollomon, Swift, and the modified Voce equations, the modified Voce equation was in the best agreement with the experimental data. The reason may have been that the strain-hardening characteristics of the alloy were different at different stages. The modified Voce equation has a saturated characteristic of strain hardening in a large strain region [30]. In the Hollomon and Swift equations, the actual *n* parameters vary in different deformation stages, and the *n* parameters are affected by temperatures, which leads to the deviation between the fitting in the whole range and the actual one to a certain extent [31].

Based on the comparisons and analysis, the modified Voce equation with a higher correlation coefficient (0.9999) and a lower absolute value of average relative error (0.42%) was selected as the constitutive model for the CHN327 alloy under the moderate temperature conditions, and the constitutive model was verified by a finite element simulation.

## 4. The Selection of Ductile Fracture Criteria

Ductile fracture is a crucial element that affects the forming properties of mold materials [32]. When the plastic deformation reaches a certain limit, the surface of the hot forging die in service may experience ductile fracture and a loss of the load-carrying capacity, which is also the primary cause of hot forging die failure. The accurate prediction of a material’s fracture has great scientific significance for the fabrication and refabrication of large hot forging dies. Compared with other indicators or models, DFCs can better consider the strain loads and nonlinear stresses in plastic deformation [33].

Considering the interaction between theoretical models and material responses, DFCs are classified into two groups. The first group is known as coupled DFCs, which incorporate damage accumulation into the constitutive equations. The other group is called uncoupled DFCs, which neglect the effects of damage on the yield surface of materials. In the uncoupled DFC group, the damage accumulation is formulated empirically or on the basis of microscopic mechanisms and various hypotheses [34]. Compared with the complex coupled DFCs, uncoupled models have been widely used in industry due to their inherent simplicity and the fact that there are fewer parameters to be evaluated experimentally [35]. In addition, the coupled criteria are more difficult to incorporate into finite element software than the uncoupled criteria [36].

In the uncoupled DFC group, damage accumulation is formulated empirically or semi-empirically with the general function in Equation (8), in terms of stress, strain, and so on, which are most relevant to the fracture initiation and propagation [37].

(8) $$\int_{0}^{{\bar{\varepsilon }}_{f}}f(\bar{\sigma },\bar{\varepsilon })d\bar{\varepsilon }\geq C_{c}$$

where ${\bar{\varepsilon }}_{f}\;$ is the fracture strain, $\bar{\sigma }$ is the von Mises equivalent stress, $\bar{\varepsilon }$ is the equivalent plastic strain, and *C_c_* is the threshold damage value of the criterion at the instance of fracture initiation.

There are various forms of DFCs based on different theories, and the applicable scope of each is different as well. According to the research basis in [10], the fracture mechanism of the CHN327 alloy at high temperatures is quasi-cleavage mixed ductile fracture. In this study, several of the most representative uncoupled DFCs were considered to predict the fracture of the CHN327 alloy, as shown in Table 6 [12].

In these equations, $\bar{\sigma }$ is the von Mises equivalent stress, $\sigma^{*}$ is the first principal tensile stress, C_1_~C_5_ are the damage limits, and $\sigma_{m}$ is the mean hydrostatic stress calculated by Equation (9):

(9) $$\sigma_{m}=\frac{\sigma_{1}+\sigma_{2}+\sigma_{3}}{3}$$

Here, $\sigma_{1}$, $\sigma_{2}$, and $\sigma_{3}$ represent the principal stresses [38].

In the uniaxial tensile test, it can be inferred that the average stress is one third of the von Mises equivalent $\bar{\sigma }\;$ or the first principal tensile stress $\sigma^{*}$ (Equation (10)).

(10) $$\sigma_{m}=\frac{1}{3}\bar{\sigma }=\frac{1}{3}\sigma^{*}$$

Then, after inserting Equation (10) into the formulas of Table 6, the simplified models are presented in Table 7 [39].

## 5. Comparison of the FE Simulation and Experimental Results

Each of the five ductile fracture criteria acted as an indicator of failure with a certain critical value that could be determined by a simulation and experiments. When the failure indicator exceeded the certain limit, cracking and fractures began. Generally, when applied to cold forming processes, the constant *C_i_* is referred to as a material constant. However, since the hot formability of nickel-based alloys is greatly affected by the temperature and strain rate, *C_i_* was no longer allowed to be considered a constant.

### 5.1. Determination of Critical Value

**Figure 6:**
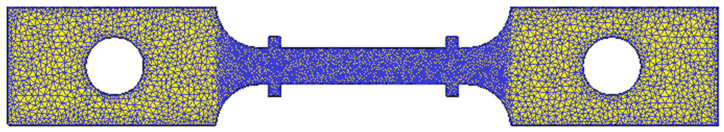
Finite element model of the tensile test.

The determination of the critical value in the ductile fracture criteria is the precondition for applying the criteria, and the accuracy of the values has a decisive effect on the accuracy of the predicted results. The critical values *C*_1_*~C*_5_ in the five DFCs could be obtained by tensile testing and a numerical simulation. In this study, a finite element model with the same size and stretching rate as the experiment was established on the DEFORM platform. As shown in Figure 6, the mesh refinement was used in the sample gauge length. The detailed procedures are given below:

(1) Obtain the fracture strain at the fracture moment from the tensile test.
(2) Incorporate the modified Voce equation of CHN327 alloy into the DEFORM-3D.
(3) Simulate the entire tension test process until the maximum principal strain reaches the value obtained in the tests, then calculate the stress–strain relationship of a point chosen from the maximum principal stress.
(4) In the five ductile fracture criteria, substitute the above stress–strain relationship for the element. Then, integration yields the material constant *C*_1_*~C*_5_.

According to the above method, the critical values of the CHN327 alloy at different temperatures and strain rates obtained by the tensile test for the five DFCs are listed in Table 8.

### 5.2. Comparison of Simulation Results with Different Ductile Fracture Criteria

**Figure 7:**
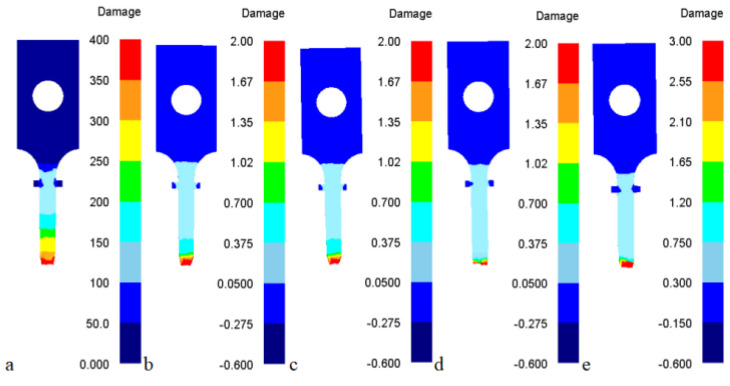
Distributions of the damage values at temperature 600 °C and strain rate 0.1 s^−1^ in the tensile test with different criteria at fracture moment: (**a**) Freudenthal model, (**b**) C&L model, (**c**) Brozzo model, (**d**) Ayada model, (**e**) R&T model.

In order to verify the accuracy of the five DFCs, the five DFCs and their corresponding critical values were substituted into the simulation software (DEFORM), comparing the fracture strain during simulation with an experiment of tensile testing. The tensile tests with strain rates of 0.1 s^−1^ and temperatures of 600 °C, 650 °C, and 700 °C, respectively, were selected as comparison objects, as shown in Table 9. The error was equal to the ratio of the fracture strain predicted by the simulation to the fracture strain after experimental stretching and then subtracted by 1 (data in parentheses). In the tensile tests by simulation, all five DFCs predicted that the fracture initiation would occur at the center of the necking section due to the high concentration of plastic deformation and stress triaxiality, as shown in Figure 7.

By comparison, it can be found that the fracture strain values predicted by the criteria of Freudenthal, C&L, Brozzo, and R&T were generally greater than the fracture strain values obtained in the experiment. In addition, only the fracture strain values predicted by the Ayada criteria were less than the experimentally obtained fracture strain values. Meanwhile, the mean error predicted by the R&T criteria was 8.9%, with the highest prediction accuracy, followed by the Ayada criteria with 14.0%. The other three criteria predicted a larger mean error and a lower prediction accuracy. The error predicted by the C&L and Brozzo criteria at 600 °C in particular, was too large to be useful for predicting the fracture evolution of the CHN327 alloy.

## 6. Conclusions

In this study, an experimental investigation and theoretical model were carried out on the ductile damage and flow stress evolution of the CHN327 nickel-based superalloy, the “skin” of the “fist-like” forging die manufactured by bimetal-gradient-layer surfacing. The major conclusions drawn from this study are as follows:

(1) Firstly, the uniaxial high-temperature tensile tests showed that the flow stress of the CHN327 alloy decreased with an increasing deformation temperature (600–700 °C) and decreasing strain rate (0.001–0.1 s^−1^). When the critical value was exceeded, fractures occurred. The fracture surface morphologies of the CHN327 alloy at high temperatures indicated that the fracture mechanism was quasi-cleavage mixed ductile fracture.
(2) Then, three classical plastic constitutive equations suitable for the CHN327 alloy were employed to establish the constitutive relationship. According to the comparison of the correlation coefficient and absolute value of the average relative error, the order of priority was as follows: *R*_modified Voce_ > *R*_Swift_ > *R*_Hollomon_; *AARE*_modified Voce_ > *AARE*_Swift_ > *AARE*_Hollomon_. Among Hollomon Swift and the modified Voce equation, the modified Voce equation had the best prediction performance under moderate temperature conditions.
(3) By simulation and comparison, except for the C&L and Brozzo models, all of the other DFCs were suitable for predicting the fracture of the CHN327 alloy during tensile tests. For all of the DFCs considered, the R&T criteria provided the most accurate predictions, whose mean error was only 8.9%, far less than the values that other models had predicted, followed by the Ayada criteria with 14.0%.

## Figures and Tables

**Table 1:** Chemical composition of CHN327 alloy designed in this study, mass/%.

C	Mn	Si	P	S	Cu	Cr	Mo	Nb + Ta	Cu	Ni
0.034	2.64	0.37	0.003	0.009	0.02	15.89	1.3	1.6	0.02	Bal.

**Table 2:** Chemical composition of ferrous welding materials RMD142 used in this study, mass/%.

C	Mn	Si	P	S	Ni	Cr	Mo	V	Fe
0.23	2.44	0.79	0.014	0.012	1.9	4.54	1.46	0.18	Bal.

**Table 3:** Chemical composition of ferrous welding materials RMD248 used in this study, mass/%.

C	Mn	Si	P	S	Ni	Cr	Mo	Al	W	V	Fe
0.25	1.61	0.72	0.013	0.004	1.5	5.51	1.69	0.21	1.04	0.24	Bal.

**Table 4:** Test scheme, the fracture strain, and the peak stress by tensile tests.

Serial Number	Temperature/°C	Strain Rate/s^−1^	The Fracture Strain	The Peak Stress/MPa
1	600	0.001	0.429	853.5
2	600	0.01	0.390	939.8
3	600	0.1	0.427	1008.1
4	650	0.001	0.337	776.5
5	650	0.01	0.365	841.7
6	650	0.1	0.388	941.8
7	700	0.001	0.332	679.3
8	700	0.01	0.380	800.1
9	700	0.1	0.410	828.2

**Table 5:** Constitutive parameters in Hollomon, Swift, and the modified Voce equations fitted to stress–strain points with full range fitting by using the original equation.

Temperature T/°C	Strain Rate	Hollomon			Swift				The Modified Voce Equation			
		*K_H_*/MPa	*n_H_*	*R*	*Ks*/MPa	*Ɛ*_0_	*n_s_*	*R*	*σ*	*b*	*k*	R
600	0.001	1285.79	0.543	0.98931	1388.75	0.0956	0.8020	0.995	254.73	38.08	−1341.43	0.9999
600	0.01	1340.81	0.477	0.98519	1454.96	0.06	0.6509	0.991	317.67	52.30	−1409.96	0.9999
600	0.1	1453.76	0.446	0.98628	1505.48	0.014	0.4950	0.987	376.36	53.32	−1542.78	0.9999
650	0.001	1227.43	0.447	0.98441	1142.12	−0.019	0.3718	0.989	337.35	34.49	−1261.64	0.9999
650	0.01	1312.92	0.456	0.97365	1198.64	−0.0286	0.3548	0.981	366.52	31.97	−1290.30	0.9999
650	0.1	1422.86	0.467	0.98796	1368.31	−0.0133	0.4184	0.989	375.24	34.04	−1425.41	0.9999
700	0.001	1100.76	0.379	0.95982	989.05	−0.0256	0.2823	0.992	400.4	24.86	−1078.54	0.9999
700	0.01	1252.39	0.523	0.98901	1252.39	−5 × 10^−17^	0.5231	0.989	282.04	28.81	−1259.64	0.9999
700	0.1	1262.26	0.521	0.98762	1227.84	−0.01307	0.4787	0.988	290.23	28.6	−1250.26	0.9999

**Table 6:** Brief summary of selected typical uncoupled DFCs.

Criteria	Formula	Background
Freudenthal	$\int_{0}^{{\bar{\varepsilon }}_{f}}\bar{\sigma }d\bar{\varepsilon }=C_{1}$	Plastic work
Normalized Cockcroft–Latham	$\int_{0}^{{\bar{\varepsilon }}_{f}}\frac{\sigma^{*}}{\bar{\sigma }}d\bar{\varepsilon }=C_{2}$	Maximum principal stress
Brozzo	$\int_{0}^{{\bar{\varepsilon }}_{f}}\frac{2\sigma^{*}}{3\left(\sigma^{*}-\sigma_{m}\right)}d\bar{\varepsilon }=C_{3}$	Effect of the mean stress on the equivalent plastic strain
Ayada	$\int_{0}^{{\bar{\varepsilon }}_{f}}\frac{\sigma_{m}}{\bar{\sigma }}d\bar{\varepsilon }=C_{4}$	Based on stress triaxiality effects on void growth neglecting void nucleation and coalescence
Rice & Tracey (R&T)	$\int_{0}^{{\bar{\varepsilon }}_{f}}\exp(\frac{3\sigma_{m}}{2\bar{\sigma }})d\bar{\varepsilon }=C_{5}$	Effect of stress triaxiality on void

**Table 7:** DFCs used in the present work.

Criteria	Formula
Freudenthal	$\int_{0}^{{\bar{\varepsilon }}_{f}}\bar{\sigma }d\bar{\varepsilon }=C_{1}$
Normalized Cockcroft–Latham	$\int_{0}^{{\bar{\varepsilon }}_{f}}\frac{\sigma^{*}}{\bar{\sigma }}d\bar{\varepsilon }=C_{2}\to {\bar{\varepsilon }}_{f}=C_{2}$
Brozzo	$\int_{0}^{{\bar{\varepsilon }}_{f}}\frac{2\sigma^{*}}{3\left(\sigma^{*}-\sigma_{m}\right)}d\bar{\varepsilon }=C_{3}\to {\bar{\varepsilon }}_{f}=C_{3}$
Ayada	$\int_{0}^{{\bar{\varepsilon }}_{f}}\frac{\sigma_{m}}{\bar{\sigma }}d\bar{\varepsilon }=C_{4}\to \frac{1}{3}{\bar{\varepsilon }}_{f}=C_{4}$
Rice & Tracey (R&T)	$\int_{0}^{{\bar{\varepsilon }}_{f}}\exp(\frac{3\sigma_{m}}{2\bar{\sigma }})d\bar{\varepsilon }=C_{5}\to 1.6487{\bar{\varepsilon }}_{f}=C_{5}$

**Table 8:** Values of damage limit obtained by the tensile test.

Serial Number	Temperature/°C	Strain Rate/s^−1^	Damage Limit of Freudenthal *C*_1_	Damage Limit of Normalized C&L *C*_2_	Damage Limit of Brozzo *C*_3_	Damage Limit of Ayada *C*_4_	Damage Limit of R&T *C*_5_
1	600	0.001	7.261	0.429	0.429	0.174	0.788
2	600	0.01	68.113	0.390	0.390	0.136	0.657
3	600	0.1	295.586	0.427	0.427	0.144	0.705
4	650	0.001	5.880	0.337	0.337	0.139	0.623
5	650	0.01	64.327	0.365	0.365	0.128	0.616
6	650	0.1	286.729	0.388	0.388	0.130	0.638
7	700	0.001	4.815	0.332	0.332	0.135	0.611
8	700	0.01	60.579	0.380	0.380	0.127	0.628
9	700	0.1	257.983	0.410	0.410	0.138	0.676

**Table 9:** The fracture strain obtained by simulation and experiment.

Serial Number	Temperature /°C	Strain Rate /s^−1^	The Fracture Strain of Freudenthal	The Fracture Strain of C&L	The Fracture Strain of Brozzo	The Fracture Strain of Ayada	The Fracture Strain of R&T	The Fracture Strain of Experiment
1	600	0.1	0.530 (24.1%)	0.77 (80.3%)	0.722 (69.1%)	0.309 (27.6%)	0.496 (16.2%)	0.427
2	650	0.1	0.477 (22.9%)	0.429 (10.6%)	0.419 (8.0%)	0.362 (6.7%)	0.413 (6.4%)	0.388
3	700	0.1	0.498 (21.5%)	0.451 (10%)	0.455 (10.9%)	0.378 (7.8%)	0.427 (4.1%)	0.41
Mean error			22.8%	33.6%	29.3%	14.0%	8.9%	

## Data Availability

The data presented in this study are not publicly available due to ongoing research in this field.

## References

[1] Zhang J., Zhou J., Tao Y., Shen L., Li M. (2015). The microstructure and properties change of dies manufactured by bimetal-gradient-layer surfacing technology. Int. J. Adv. Manuf. Technol..

[2] Altan T., Lilly B., Yen Y.C. (2001). Manufacturing of dies and molds. CiRP Ann..

[3] Guoji L., Wang J., Zhao X., Wang J. (2009). Study on mechanical properties and microstructure of gradient functional layer prepared by CO_2_ surfacing welding with electromagnetic stir. Acta Met. Sin..

[4] Sun G. (2006). New technology of repair of forging die by built-up welding. China Met. Equip. Manuf. Technol..

[5] Liu Y.F., Xia Z.Y., Han J.M., Zhang G.L., Yang S.Z. (2006). Microstructure and wear behavior of (Cr, Fe) 7C3 reinforced composite coating produced by plasma transferred arc weld-surfacing process. Surf. Coat. Technol..

[6] Lu S., Zhou J., Zhang J. (2015). Optimization of welding thickness on casting-steel surface for production of forging die. Int. J. Adv. Manuf. Technol..

[7] Shen L., Zhou J., Xiong Y.B., Zhang J.S., Meng Y. (2018). Analysis of service condition of large hot forging die and refabrication of die by bimetal-layer weld surfacing technology with a cobalt-based superalloy and a ferrous alloy. J. Manuf. Process..

[8] Gao F., Zhou J., Zhou J., Shen L., Zhang J., Tao Y., Li M. (2017). Microstructure and properties of surfacing layers of dies manufactured by bimetal-gradient-layer surfacing technology before and after service. Int. J. Adv. Manuf. Technol..

[9] Yang S.B., Dong W., Xu X.C., Li T. (2012). Research actuality and developmentof the Fe-Cr-C wearresistant hardfacing alloy system. Mater. Rev..

[10] Xia Y., Jin L., Cheng Q., Tong J., Zhang K., Zhang J. (2019). A comparative study on the microstructures and mechanical properties between surfacing nickel-based superalloy and surfacing cobalt-based superalloy. Mater. Res. Express.

[11] Xia Y., Chen Y., Peng M., Teng H., Zhang X. (2022). A Comparative Study on the Microstructures and Mechanical Properties of Two Kinds of Iron-Based Alloys by WAAM. J. Wuhan Univ. Technol. Mater. Sci. Ed..

[12] Li H., Fu M.W., Lu J., Yang H. (2011). Ductile fracture: Experiments and computations. Int. J. Plast..

[13] Mei Z., Gu C., Jiang Z., Hu L., Yang H. (2009). Application of ductile fracture criteria in spin-forming and tube-bending processes. Comput. Mater. Sci..

[14] Lou Y., Huh H. (2013). Prediction of ductile fracture for advanced high strength steel with a new criterion: Experiments and simulation. J. Mater. Process. Technol..

[15] Wang H.J., Wu Y.Z., Wang H.C., Sun Y.Z., Wang G. (2011). Design method and verification for long life hot forging die. Mater. Res. Innov..

[16] Speidel M.O. (1999). Wear and corrosion resistance of PM tool steels. Met. Powder Rep..

[17] Cui C.Y., Xia C.D., Cui X.G., Zhou X., Ren D., Wang Y.M. (2015). Novel morphologies and growth mechanism of Cr_2_O_3_ oxide formed on stainless steel surface via Nd: YAG pulsed laser oxidation. J. Alloy. Compd..

[18] Xia Y.F., Teng H.H., Cheng Q. (2020). Properties and flow stress model of nickel-based alloy by wire arc additive manufacturing. Trans. Mater. Heat Trement.

[19] Lin J., Mohamed M., Balint D., Dean T.A. (2014). The development of continuum damage mechanics-based theories for predicting forming limit diagrams for hot stamping applications. Int. J. Damage Mech..

[20] Xiao W., Wang B., Wu Y., Yang X. (2018). Constitutive modeling of flow behavior and microstructure evolution of AA7075 in hot tensile deformation. Mater. Sci. Eng. A.

[21] Bhaduri A. (2018). Mechanical Properties and Working of Metals and Alloys.

[22] Guo J., Zhao S., Murakami R., Fan S. (2013). Modeling the hot deformation behavior of Al alloy 3003. J. Alloy. Compd..

[23] Hertelé S., De Waele W., Denys R. (2011). A generic stress—Strain model for metallic materials with two-stage strain hardening behaviour. Int. J. Non-Linear Mech..

[24] Swift H.W. (1952). Plastic instability under plane stress. J. Mech. Phys. Solids.

[25] Voce E. (1948). The relationship between stress and strain for homogeneous deformation. J. Inst. Met..

[26] Guo J.H., Zhao S.D., Yan G.H., Wang Z.B. (2013). Novel flow stress model of AA 4343 aluminium alloy under high temperature deformation. Mater. Sci. Technol..

[27] Christopher J., Choudhary B.K., Samuel E.I., Mathewm D., Jayakumar T. (2012). Tensile stress—Strain and work hardening behaviour of P9 steel for wrapper application in sodium cooled fast reactors. J. Nucl. Mater..

[28] Shokry A., Gowid S., Youssef S.S. (2022). Modeling the flow behavior of Haynes 214 superalloy during hot deformation using mathematical and artificial intelligence-based models. Mater. Today Commun..

[29] Shokry A. (2019). On the constitutive modeling of a powder metallurgy nanoquasicrystalline Al_93_Fe_3_Cr_2_Ti_2_ alloy at elevated temperatures. J. Braz. Soc. Mech. Sci. Eng..

[30] Jun C.A.O., Li F., Sun Z. (2017). Tensile stress—Strain behavior of metallic alloys. Trans. Nonferrous Met. Soc. China.

[31] Samuel K.G. (2005). Limitations of Hollomon and Ludwigson stress—Strain relations in assessing the strain hardening parameters. J. Phys. D Appl. Phys..

[32] Lassance D., Fabregue D., Delannay F., Pardoen T. (2007). Micromechanics of room and high temperature fracture in 6xxx Al alloys. Prog. Mater. Sci..

[33] Ma H., Xu W., Jin B.C., Shan D., Nutt S.R. (2015). Damage evaluation in tube spinnability test with ductile fracture criteria. Int. J. Mech. Sci..

[34] Zadpoor A.A., Sinke J., Benedictus R. (2009). Formability prediction of high strength aluminum sheets. Int. J. Plast..

[35] Xu W., Wu H., Ma H., Shan D. (2018). Damage evolution and ductile fracture prediction during tube spinning of titanium alloy. Int. J. Mech. Sci..

[36] Xue Z., Pontin M.G., Zok F.W., Hutchinson J.W. (2010). Calibration procedures for a computational model of ductile fracture. Eng. Fract. Mech..

[37] Wierzbicki T., Bao Y., Lee Y.W., Bai Y. (2005). Calibration and evaluation of seven fracture models. Int. J. Mech. Sci..

[38] Hashemi R., Abrinia K. (2014). Analysis of the extended stress-based forming limit curve considering the effects of strain path and through-thickness normal stress. Mater. Des..

[39] Heidari A., Ghassemi A., Atrian A. (2020). A numerical and experimental investigation of temperature effects on the formability of AA6063 sheets using different ductile fracture criteria. Int. J. Adv. Manuf. Technol..

